# Nonsignificance misinterpreted as an effect’s absence in psychology: prevalence and temporal analyses

**DOI:** 10.1098/rsos.242167

**Published:** 2025-03-19

**Authors:** Stephen Lee Murphy, Raphael Merz, Linda-Elisabeth Reimann, Aurelio Fernández

**Affiliations:** ^1^ Ghent University, Ghent, Flanders, Belgium; ^2^ Ruhr University Bochum, Bochum, North Rhine-Westphalia, Germany; ^3^ University of Münster, Münster, North Rhine-Westphalia, Germany; ^4^ University of Navarra, Pamplona, Navarre, Spain

**Keywords:** non-significance, metascience, metaresearch, misinterpretation, statistics

## Abstract

Nonsignificant findings in psychological research are frequently misinterpreted as reflecting the effect’s absence. However, this issue’s exact prevalence remains unclear, as does whether this issue is getting better or worse. In this pre-registered study, we sought to answer these questions by examining the discussion sections of 599 articles published across 10 psychology journals and three time points (2009, 2015 and 2021), and coding whether a nonsignificant finding was interpreted in such a way as to suggest the effect does not exist. Our models indicate that between 76% and 85% of psychology articles published between 2009 and 2021 that discussed a nonsignificant finding misinterpreted nonsignificance as reflecting no effect. It is likely between 54% and 62% of articles over this time period claimed explicitly that this meant no effect on the population of interest. Our findings also indicate that only between 4% and 8% of articles explicitly discussed the possibility that the nonsignificant effect may exist but could not be found. Differences in prevalence rates over time were nonsignificant. Collectively, our findings indicate this interpretative error is a major problem in psychology. We call on stakeholders with an interest in improving psychological science to prioritize tackling it.

## 1. Introduction

Why should any correlation coefficient be exactly .00 in the population? Why should we expect the ratio of males to females be exactly 50:50 in any population? Or why should different drugs have exactly the same effect on any population parameter? A glance at any set of statistics on total populations will quickly confirm the rarity of the null hypothesis in nature. [1, p. 426]

Nonsignificant findings in the null hypothesis significance testing tradition are commonplace in psychological research [2]. This is due both to the use of one-sided statistical tests (effects in the direction opposite to that hypothesized are always nonsignificant) and research having less than perfect statistical power to find what are theorized to often be small-to-medium effects [3]. When a nonsignificant finding results, it is tempting to conclude that it means the investigated effect does not exist. However, this would be incorrect, for nonsignificance indicates only that the data are unsurprising under the null hypothesis [4]. In other words, claiming from a nonsignificant finding that ‘an effect does not exist’, or that ‘experimental conditions exert the same impact’, is problematic because it is highly likely that a larger sample would have rendered the nonsignificant effect significant. Moreover, a true population effect nearly *always* exists [1,5]—generally, only the effects direction and magnitude need determining [6]. The reality that nonsignificance does not represent the effect’s absence has been stressed repeatedly over the past decades [1,6–9]. Tukey [8] made this reality particularly evident: ‘It is foolish to ask “Are the effects of A and B different?” They are always different—for some decimal place’ (p. 100).

Despite ubiquitous educational efforts on this topic [10], in their published work, psychological researchers frequently use language that conveys that nonsignificance means that an effect does not exist [2,11]. For instance, upon finding a nonsignificant effect, Boudreaux & Ozer [12] concluded that goal conflict has no impact on goal attainment, Klaperski *et al*. [13] reported no relationship between physiological and psychological reactions to stress, Townsend *et al*. [14] argued that ‘women’s chronic perceptions of sexism were unrelated to their cortisol changes when there were clear identity-safe cues’ (p. 160), and Yoo *et al*. [15] reported that partners’ expressed anger does not influence relationship satisfaction when communal motivation is high. That researchers often discuss nonsignificant effects in this way is problematic, for many readers will take it literally that nonsignificance means the effect is absent. Moreover, this practice is likely to discourage researchers that take the message literally from investigating the effect and will surely disrupt effective policymaking. Why do psychological researchers often convey this incorrect message, then? The most obvious explanation is that they truly believe that nonsignificance means that there is no effect, and correspondingly, that nil effects (i.e. effects that are *exactly* zero [1]), are fairly prevalent eventualities in nature. However, what also may exert an influence is the culture in psychology that applauds flashy, unintuitive results [16], and that a binary ‘effect-no effect’ communication style corresponds with the crude but convenient way in which researchers probably discuss effects outside of the scientific arena [7].

Various research efforts have attempted to better understand the prevalence of this issue by examining the published psychology literature and uncovering what percentage of articles that found a nonsignificant result equated nonsignificance with an effect’s absence. Finch *et al*. [17] did this by examining the first 30 articles published in the *Journal of Applied Psychology* for the years 1940, 1955, 1970, 1985 and 1999, Hoekstra *et al*. [11] did this by examining 242 articles published between the years 2002 and 2004 from *Psychonomic Bulletin and Review*, while Aczel *et al*. [2] did this by examining the abstracts of 132 articles published in 2015 in *Psychological Science*, *Psychonomic Bulletin & Review* and *Journal of Experimental Psychology: General*. They found that 38%, 63% and 72% of articles, respectively, misinterpreted nonsignificance as reflecting an effect’s absence. Moreover, Aczel *et al*. [2] found that 67% of these misinterpretations communicated as if the sample was the focus (e.g. ‘groups *were* indifferent’). These sample-based interpretations—which are primarily identifiable from their use of the past instead of the present tense—are incorrect because these inferential tests were employed to learn something about the overarching population. These findings are valuable. Alongside alternative approaches to understanding the prevalence of this issue (e.g. scenario-based approaches that get researchers to state whether stock conclusions from statistical test output are correct, see [18]), they collectively indicate that nonsignificance may be misinterpreted as an effect’s absence by a large percentage of, or even most, published psychology articles that employ null hypothesis significance testing. These findings are also valuable for they inform stakeholders interested in improving psychological research (e.g. journal editors, OSF) of what issues should be prioritized.

Nevertheless, more research is needed to firm up the *real* prevalence of this issue. First, because two key studies examined articles from more than two decades ago [11,17]. Considering the many published articles over the past few decades that focused on improving the statistical literacy of psychological researchers [19–21], it is possible the prevalence of this issue is very different today or in the recent past relative to the more distant past. Research that can examine prevalence rates from the more recent past would thus prove highly informative. Second, because extant research focused only on a handful of psychology journals. Research that can examine this issue across many more psychology journals, preferably differing markedly on some marker of quality, would provide more representative prevalence estimates. Third, because the method employed by some extant research had limitations. Aczel *et al*. [2] examined article abstracts only (abstract space limitations discourage discussion of nonsignificant findings), Finch *et al*. [17] coded incorrect interpretations of nonsignificance as correct if statistical output underpinning the interpretation was not spatially proximal to it, while both Aczel *et al*. [2] and Finch *et al*. [17] coded statements that used softer language to suggest that nonsignificance means the effect is absent (e.g. ‘the groups were *similar*’ or ‘results suggest that age *barely impacts* self-regulation’) as correct interpretations of nonsignificance. All of these methodological choices probably biased prevalence estimates downwards, thus research free of these limitations would better reveal the problems’ true scale. Finally, because no research in the past decade or so has collected data to formally model whether the prevalence of this issue has increased or decreased over time. While such insights can be gleaned by comparing the findings of two or more studies focused on different temporal periods, it is possible the various methodological differences that exist between studies (e.g. coding strategy) may underlie differences in prevalence estimates.

### 1.1. Present study

Accordingly, the primary aim of the present study is to reinvestigate how prevalent it is for published psychology research employing null hypothesis significance testing to misinterpret nonsignificance as reflective of an effect’s absence. We do this by examining 599 articles that mention a nonsignificant finding in their discussion section, that are published across 10 psychology journals that differed markedly on their SCImago Journal Rank (a putative marker of quality), in the years 2009, 2015 and 2021, and then coding whether a nonsignificant finding from that discussion section was interpreted as an effect’s absence. This research will provide a better insight into the scale of this issue given (i) the sample of articles examined is more representative of all psychology articles published over the past few decades, (ii) in line with Aczel *et al*. [2] we delineate how many misinterpretations seem to be sample and population-focused, respectively, and (iii) we overcome methodological issues present in extant literature that probably biased prevalence estimates downwards (e.g. ‘softer’ statements that claim effects ‘are similar’ or ‘essentially the same’, are coded in the present study as having communicated the effects absence). For the readers’ interest, we highlight how many of the articles coded as having misinterpreted nonsignificance were coded as such from the presence of soft language *only*. Inferential insights in relation to this content are also provided.

The second key study aim is to examine whether the prevalence of this interpretative error in the psychological literature is increasing or decreasing over time, by contrasting prevalence estimates between the examined article publication years. Considering the many efforts over the past decade or so to improve psychologists’ statistical literacy, we hypothesize that in 2009 relative to 2015 and 2021, and in 2015 relative to 2021, more published psychology articles that discussed a nonsignificant finding misinterpreted nonsignificance as reflecting no effect. We additionally test these hypotheses specifically in relation to the presence of population-based misinterpretations, whereby study authors make it particularly clear that their statement refers to the population of interest (e.g. ‘men and women *do not* differ in their self-regulatory capacities’).

Our final study aims are twofold. First, we hope to elucidate whether articles that are more impactful (i.e. that have more citations) are less likely to misinterpret a nonsignificant finding as reflecting an effect’s absence. This is possible, for the effort and care that one has to put in to deliver a high-quality piece may also manifest in greater congruence between statistical output and conclusions. That stated, what may work against this mechanism is any process whereby authors elevate their article’s impact by embellishing their findings—something authors are known to do [16]—hence why empirical research is needed. Second, we aim to uncover what percentage of psychology articles that mention a nonsignificant finding made it particularly explicit that it could have been an *effect could not be found* issue, rather than an *effect does not exist* issue. That nonsignificance reflects an inability to find the issue is highly probable, thus it would be valuable to document how often articles explicitly discuss this as a possibility. Moreover, articles that *do* discuss this as a possibility arguably best ensure readers leave with an accurate take-home message—that the investigated effect very likely exists, it is just a matter of uncovering its direction and magnitude.

## 2. Disclosures

### 2.1. Preregistration

This study was pre-registered: https://doi.org/10.17605/OSF.IO/ASGYH.

### 2.2. Data, materials and online resources

All data, R code and materials are available at our OSF page (https://osf.io/qvzr8/).

### 2.3. Reporting

We report all data exclusions and measures collected in this study and specify clearly which analyses are confirmatory and exploratory. This study deviated from our preregistration in five places: (i) we planned to determine the prevalence of nonsignificance misinterpretations in psychology articles over the examined period by—like prior research [2]—simply counting articles that did and did not make this error within the sample. However, we later recognized that it is more appropriate to investigate this with an inferential test, hence, we instead opted to use a multi-level binomial regression to investigate this issue; (ii) we pre-registered that we would investigate some research questions (e.g. whether the prevalence of nonsignificance misinterpretations varies across time points) with a multi-level model with fixed slopes. However, we recognized later that, theoretically, a random slope made more sense, for any change in misinterpretation prevalence over time would very likely vary at level 2 across journals. Nevertheless, although all analyses include a fixed *and* random slope, we can confirm no meaningful differences exist in results had the slope remained fixed; (iii) we planned exploratory analyses to additionally provide likelihood ratios for our results (for example of what insights these can provide, see [22]). However, given in most instances our confidence intervals are quite narrow, we felt this addition was not particularly insightful; (iv) we planned to explore whether significant effects in articles that also found nonsignificant effects and interpreted them as reflecting the effect’s absence were similar in size to the considered nonsignificant effects. This analysis was planned to challenge a claim researchers could plausibly make that because nonsignificance signals that the effect *may* be smaller than anticipated, the effect probably has a magnitude that is below the threshold the researchers consider to be of interest, hence why they employed ‘no effect’ terminology. By showing that even very small *significant* effects are very rarely described with ‘no effect’ terminology, we hoped to weaken this claim. However, we felt we could just as easily weaken this claim without additional analyses, by highlighting that not one of the articles we examined specified the smallest effect size of interest and that only a handful of significant effects were described with ‘no effect’ terminology; and (v) if a statement extracted from an article was coded differently by the researchers assigned to it, the two lead researchers met to decide which code fit best. This represents a deviation from our preregistration which stated the two researchers that initially allocated the codes would determine the final code. We deviated due to a larger number of coding disagreements than anticipated, and thus the considerable time and training demands that would have ensued to complete this research step across four researchers.

## 3. Methods

### 3.1. Screening procedure

We aimed to examine 600 articles published across 10 psychology journals at three separate time points (2009, 2015 and 2021) that mentioned in their discussion section at least one of their nonsignificant results. This equated to 200 articles per year or 20 articles *per journal* per year. This sample size was a trade-off between our aim to get as much data as possible to investigate prevalence rates and temporal effects, and the considerable time demands of coding many more articles. This sample size was also informed by prior literature investigating this topic: Hoekstra *et al*. [11] and Aczel *et al*. [2] used samples of 242 and 132 articles, respectively; hence we wanted to exceed these across more journals to better ensure our study represented a valuable empirical contribution. The selected journals were *Psychological Science*, *Personality and Social Psychology Bulletin*, *Journal of Personality*, *British Journal of Social Psychology, European Journal of Social Psychology*, *Journal of Social and Personal Relationships, Motivation and Emotion*, *International Journal of Behavioral Development*, *The Journal of Social Psychology* and the *Journal of Community Psychology*. These journals were selected because they published enough articles each year to enable us to meet our 20 articles per journal per year target, and because they varied markedly in their SCImago Journal Rank (see https://www.scimagojr.com/), which represents a crude measure of journal quality. We recorded this rank in July 2022.

Article selection from these journals was systematic. The first article published within each of the 10 journals at each of the three time points was assessed and included/excluded in/from our study according to pre-specified criteria (detailed in the next paragraph). Following, the last published article from each of these journals for each year was similarly assessed and included/excluded, then the second published article, then the second last published article, etc. until 20 articles were included in our study.

Four researchers were involved in this screening step. First, the article title and abstract were checked to confirm the article was not a review (e.g. narrative, systematic, meta-analysis, etc.), editorial, corrigendum/erratum, correspondence/reply, retraction or something of a similar nature, as original empirical research was our primary focus. If not excluded at this step, the full article was examined and included in our analyses if *all* of the following criteria were met: (i) the article contained a distinct ‘results’ and ‘discussion’ section (sections that served the same purpose but that were termed differently were also included). These criteria aided systematic selection of article statements and were met by most articles; (ii) the article contained at least one *p*-value in the results section (in a multi-study paper, this could be in *any* results section). This confirmed the article used null hypothesis significance testing. Please note, we did not include the handful of articles that included *p*-rep instead of *p*-values (see [23] for details); (iii) article discussion section (or General Discussion section in a multi-study paper) mentioned a nonsignificant effect *that clearly originated from that articles own research*; (iv) article did not contain Bayesian analyses. A total of 599 articles were included in our analyses after this step (*British Journal of Social Psychology* for the year 2009 could only provide 19 and not 20 articles).

Four researchers then scanned the discussion section (or General Discussion section, in a multi-study paper) of each included article and highlighted *all* text where study authors mentioned or interpreted a nonsignificant effect. Where a researcher could not find a nonsignificant effect (due to an error in not excluding an article at prior step), they followed the systematic approach to article selection to replace the article. Each researcher highlighted a text amount from each article that they felt provided sufficient context to the nonsignificance statement. If only one text-block/statement was highlighted in an article, we coded whether their nonsignificance interpretation was incorrect or not in the next step. If multiple statements were highlighted, the researcher selected that statement (for subsequent coding/appraisal) which they felt best revealed the authors’ understanding of what nonsignificance meant. In articles that contained statements that did and did not misinterpret nonsignificance, very often it was the incorrect interpretation statement that was considered the most reflective of the authors’ understanding; generally, these statements were more revealing (e.g. ‘the effect was nonsignificant’—no misinterpretation, versus ‘This means that men and women do not differ in trait self-control’—misinterpretation). We examined the objectivity/reliability of this step by having two researchers independently extract and code statements from the same 60 articles. This article sample was systematically chosen: after articles were ordered by journal and year, every ninth article was selected.

After extracting 659 statements containing an interpretation of nonsignificance (one statement from 539 articles, two statements from 60 articles), two researchers, blind to any article-specific information (e.g. journal and publication year), independently coded them for various criteria (see §3.2 for details). Each statement was coded as either (i) not having misinterpreted nonsignificance as reflecting no effect (§3.2.1), (ii) having misinterpreted nonsignificance as there being no effect in the sample (§3.2.2), and (iii) having misinterpreted nonsignificance as there being no effect in the population (§3.2.3). If a statement was coded as a No Misinterpretation, we additionally coded whether that statement contained (i) the term ‘nonsignificant’, or a term similar in type (e.g. ‘not significant’, ‘nonsignificantly’, etc.), (ii) terminology indicating that the effect could not be *found* (e.g. ‘we did not find effect’, ‘analyses did not reveal an effect’), and (iii) a more detailed discussion citing the possibility that the nonsignificant effect simply may not have been found (e.g. ‘This nonsignificant finding could reflect a statistical power or measurement sensitivity issue’). If a statement was coded as either a Sample or Population Misinterpretation, we additionally coded whether the statement contained, with reference to the nonsignificant effect (i) minimal effect terminology (e.g. ‘the response was similar between conditions’, ‘minimal differences existed between groups’, etc.), (ii) no effect terminology (e.g. ‘the response was the same between conditions’, ‘no differences existed between groups’, etc.), (iii) past tense terminology (‘effects *were* different’), (iv) present tense terminology (‘effects *are* different’), and (v) Sample Terminology—that is, terminology that only really makes sense if the sample is the focus (‘self-control in conditions A and B is indifferent’). We additionally coded these statement characteristics to gain greater insight into the nature of statements that do and do not misinterpret nonsignificance, to answer various exploratory research questions and to help objectively allocate statements to their (mis)interpretation category (see §3.2.2 for details).

Using two researchers enabled the examination of the objectivity/reliability of this coding step. In the handful of cases where a researcher was not sure the statement referred to a nonsignificant finding, they extracted and coded another statement from the same article. If that article did not contain any more nonsignificance statements, the researcher systematically chose another article and extracted and coded an appropriate statement. If after all statements were coded any two researchers disagreed on a statement code, the two lead authors discussed it and agreed upon a final code. If a researcher disagreed with the code allocated by another researcher to either of the two statements extracted from the 60 reliability articles, the two lead researchers discussed and agreed upon (i) which of the two statements was most reflective of the authors’ understanding of what nonsignificance means, and (ii) what code should be applied to the chosen statement.

### 3.2. Coding categories

#### 3.2.1. No Misinterpretation

An article was coded as not having misinterpreted nonsignificance as indicating the effect’s absence if there was no suggestion of this in the extracted statement. Thus, if the article simply mentioned that an effect was nonsignificant, or that an effect could not be found, the statement was coded as a No Misinterpretation. An article was also coded as a No Misinterpretation if in the extracted statement there was a more detailed recognition that nonsignificance may simply reflect an effect that could not be found.

Example statements include: ‘The analysis did not show a significant effect of the intervention’, ‘The identified effect was nonsignificant’ and ‘We were not able to find a difference. There are various explanations for this nonsignificant effect—it may be that no effect exists, but it is also possible the effect exists, but we did not have the statistical power to find it’.

#### 3.2.2. Sample Misinterpretation

An article was coded as a Sample Misinterpretation if the extracted statement (i) indicated that nonsignificance reflected the effect’s absence, and (ii) indicated that the sample was the focus of this interpretation. Criterion (i) could be identified from language that suggested the effect does not exist, rather than simply an effect that could not be found. Criterion (ii) was identified if only past-tense language (so, no present-tense language) was used in relation to the nonsignificant effect, or, if the language used in relation to the nonsignificant effect reflecting sample terminology, that is, language highly incongruent with the population (e.g. if a statement contains the phrase ‘Conditions A and B do not differ’, then the sample rather than the population seems the focus, even though past-tense language is not used).^1^

Example statements include: ‘There was no difference between participants in the intervention group and the control group’, ‘The experimental group was essentially the same as the control group on this task’, and ‘Effects across conditions were similar’.

#### 3.2.3. Population Misinterpretation

An article was coded as a Population Misinterpretation if the extracted statement (i) indicated that nonsignificance reflected the effect’s absence, and (ii) indicated that the population was the focus of this interpretation. Criterion (i) could be identified from language that suggested the effect does not exist, rather than simply an effect that could not be found. Criterion (ii) was typically identified by the use of present- instead of past-tense language, or the use of language that was highly congruent with the population.

Example statements include: ‘The results establish that the social media intervention has no effect on wellbeing’, ‘Men do not have more self-control than women’ and ‘Cognitive decline does not result between the ages of 40 and 60’.

### 3.3. Analytical procedures and strategy

#### 3.3.1. Data preparation and analyses

In R [24], R Markdown [25] and R Studio [26], we organized and cleaned the data with the packages dplyr [27], readr [28], readxl [29], tidyr [30] and forcats [31], we analysed the data with the packages lme4 [32] and lmerTest [33], we visualized the results with the packages ggplot2 [34], ggdist [35], scales [36], colorspace [37], ggtext [38] and stringr [39], we rendered the final manuscript with the packages tinytex [40], papaja [40], knitr [41], qpdf [42] and pdftools [43], and we used the package trackdown [44] to aid author collaboration.

Our data were considered hierarchical, with articles (level 1) clustered within journals (level 2). This was supported from a multi-level binomial regression model with our binary misinterpretation/no misinterpretation variable as the dependent variable, and with no predictors: 2.79% of the variance was explained at level 2. To ensure standard errors are not systematically underestimated, we investigate prevalence estimates and all but one hypothesis using multi-level binomial regression [45]. To investigate whether more impactful articles contain fewer misinterpretations, a regular multi-level regression is used. To investigate the prevalence of articles that misinterpret nonsignificance as the effect’s absence, and the prevalence of articles that make Population Misinterpretations specifically, we code two variables as 1 if the article made this error, and 0 if it did not. These variables were then entered as dependent variables in a model with no predictors that used (i) all data, and data from the year (ii) 2009, (iii) 2015, and (iv) 2021, with the intercept representing the prevalence estimate. The same coding and modelling approach was used in relation to whether an article did or did not (i) use only soft terminology when claiming nonsignificance reflects no effect, and (ii) discuss explicitly that nonsignificance may simply reflect an *inability to find* the issue (rather than a *no effect* issue).

To investigate whether articles that misinterpreted nonsignificance, or specifically, made a Population Misinterpretation, were becoming more prevalent over time, dummy variables for each publication year were created and modelled with a random intercept and slope (we considered it likely that any change of prevalence over time would vary across journals). Odds ratios were then calculated and reported in our results. To test the same hypotheses with a growth curve model, we entered as a predictor the articles’ year of publication, coded as 0, 1 and 2, for the years 2009, 2015 and 2021, respectively. Finally, to investigate whether articles that misinterpreted nonsignificance, and specifically, made a Population Misinterpretation, are more or less impactful than articles that did not make such an error, we ran a multi-level linear regression with a random intercept and slope, with citation count as the dependent variable, year of publication as a control variable, and as a predictor the binary variable specifying whether or not the article made the focal interpretation error. Two-tailed tests were used for all analyses, with alpha set at 0.05.

## 4. Results

### 4.1. Coding validity and reliability

Analyses revealed that researchers agreed upon whether a statement represented a No Misinterpretation or a Sample/Population Misinterpretation in 78.80% of instances, and agreed upon whether a statement represented a No Misinterpretation, Sample Misinterpretation and Population Misinterpretation, in 58.26% of instances. Analyses of the 60 reliability articles—where two independent researchers extracted statements from the same article—revealed that researchers agreed upon whether an article represented a No Misinterpretation or a Sample/Population Misinterpretation in 70% of instances, and agreed upon whether a statement represented a No Misinterpretation, Sample Misinterpretation and Population Misinterpretation, in 50% of instances.^2^

Analyses also revealed that when both coding researchers agreed that a statement should be coded as a No Misinterpretation, they agreed that it contained nonsignificance terminology, ‘did not find’ terminology and a more explicit discussion of nonsignificance as an effect that probably could not be found, in 94.32%, 73.86% and 77.27% of instances, respectively. When both coding researchers agreed that a statement should be coded as either a Sample or Population Misinterpretation, they agreed that it contained soft ‘no effect’ language (e.g. ‘groups are *similar*’), clear-cut ‘no effect’ language (e.g. ‘groups are *the same*’), past tense, present tense, and sample terminology, in 90.77%, 81.79%, 93.14%, 67.81% and 80.74% of instances, respectively.^3^

Low inter-rater reliability scores resulted in large part due to many more articles than anticipated using vague terminology when discussing nonsignificant effects. To overcome this reliability issue, the two lead authors—who were most heavily involved in all study aspects—discussed in detail each disagreed-upon statement to arrive at final codes. Importantly, our coding demarcation criteria were refined: it was agreed that statements that included the terms *predict*, *relate* and *associate* (e.g. ‘X did not predict/relate to/associate with Y’) should generally be regarded as the author claiming nonsignificance means there is no effect (and thus would be coded as a Misinterpretation).^4^ It was also agreed that statements that included the terms *find* and *reveal* (e.g. ‘we did not find an effect’, ‘an effect was not revealed’) should generally be regarded as the author claiming nonsignificance means the effect could not be found (and thus would be coded as a No Misinterpretation). All statements and applied codes are made available at our OSF page: https://osf.io/qvzr8/.

### 4.2. Confirmatory analyses

#### 4.2.1. Nonsignificance misinterpretation prevalence

**Figure 1.**
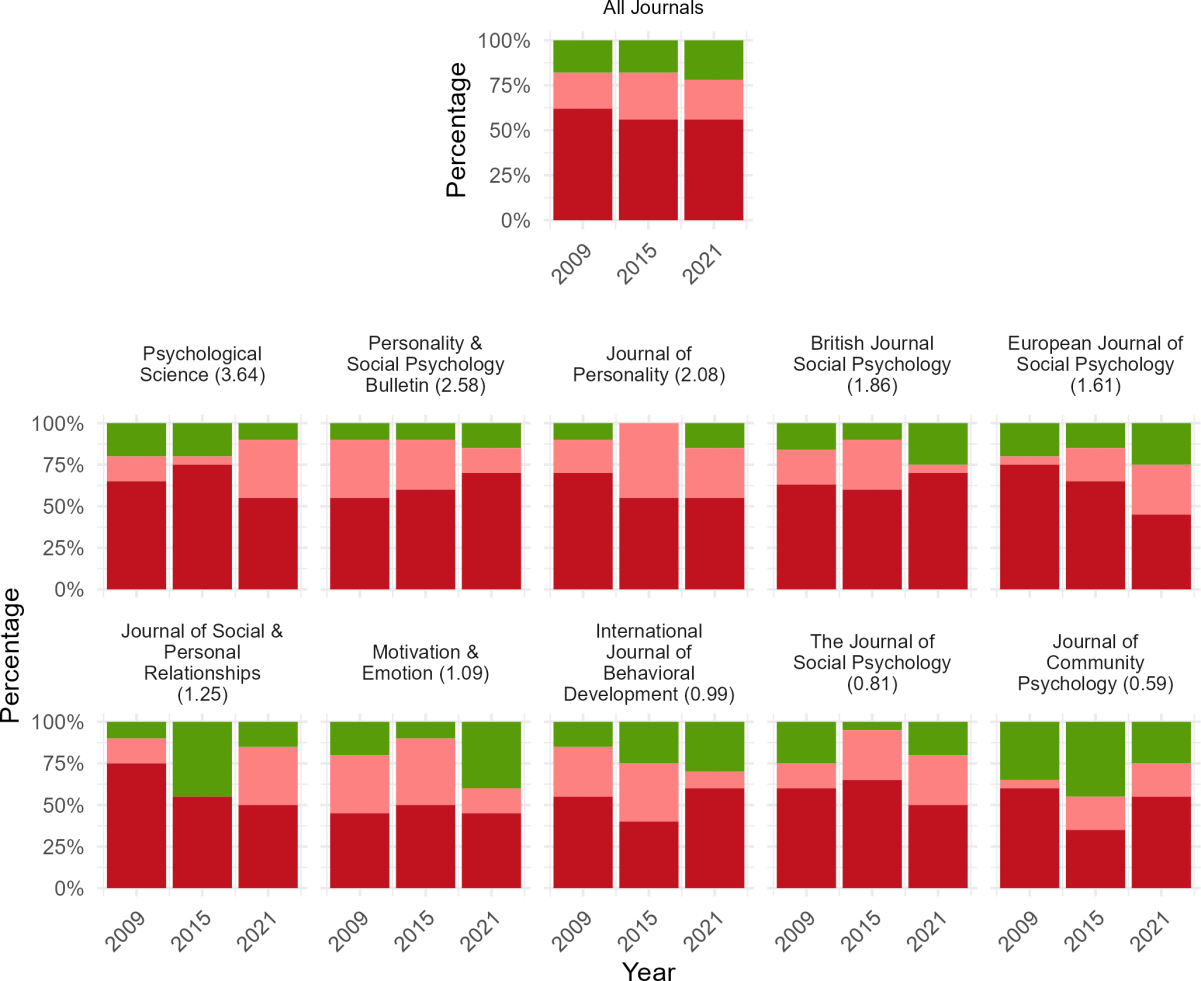
Prevalence of articles that misinterpreted nonsignificance as reflecting no effect for 10 psychology journals for the years 2009, 2015 and 2021. *Note*. Green bars represent articles that did not misinterpret nonsignificance as reflecting no effect. Light and dark red represent articles misinterpreted nonsignificance as reflecting no effect in the sample and population, respectively. ‘All Journals’ shows prevalence rates across the 10 journals depicted. The number in brackets after each journal name depicts each journal’s SCImago Journal Rank Indicator. This represents a crude measure of journal quality.

Table 1 depicts some of the statements concerning a nonsignificant finding coded in this study, along with their associated codes. Figure 1 depicts how prevalent it was for published research across 10 psychology journals in the years 2009, 2015 and 2021, to misinterpret nonsignificance as reflecting no effect. Multi-level binomial regression analysis revealed that 80.91% (95% CI: 76.17–84.90%) of all psychology articles that discussed a nonsignificant effect across the examined years may have made this interpretation error. Breaking this down, our models reveal that 81.91% (95% CI: 75.94–86.66%), 84.55% (95% CI: 73.42–91.55%) and 78% (95% CI: 71.73–83.21%) of all psychology articles for the year 2009, 2015 and 2021, respectively, probably misinterpreted nonsignificance as reflecting no effect. Fine-grained analyses indicate that 57.93% (95% CI: 53.93–61.82%) of all psychology articles that discussed a nonsignificant effect over the examined time period may have made a Population Misinterpretation. Breaking this down, our models suggest that 62.31% (95% CI: 55.38–68.77%), 56.01% (95% CI: 48.89–62.90%) and 55.50% (95% CI: 48.55–62.24%) of all psychology articles in the year 2009, 2015 and 2021, respectively, may have made a Population Misinterpretation.

**Table 1.** Examples of extracted statements and applied codes.

article	statement	applied code
Falomir-Pichastor *et al.* [46]	‘the observed findings suggest that high identifiers are overall more group-oriented than low identifiers (i.e. no significant effects were observed for low identifiers as a function of ingroup norm and ingroup threat).’	No Misinterpretation
Luhmann *et al.* [47]	‘however, a third main result was that for the majority of the investigated event domains and psychological outcomes, none of the crucial group comparisons met our thresholds for statistical significance and minimum effect size, particularly for mean-level change and for the Big Five. Of course, a nonsignificant effect does not mean that there is no effect; and it is possible that effects could have been detected if not for the specifications and limitations of our research design (see below). The more likely explanation, however, is that many missed events (and many life events for which we also did not find any significant effects) have little to no association with changes in the psychological outcomes investigated here.’	No Misinterpretation
Asselmann & Specht [48]	‘third, there was little evidence that the effects on personality around the beginning and ending of working life varied by gender, age, or employment status. These findings suggest that mean-level personality changes in the years before, during, and after these transitions did not differ between women and men, between younger and older individuals, and between people who were and were not employed full-time in the first job of working life or last year before retirement.’	Sample Misinterpretation
Morton *et al.* [49]	‘in both studies, we measured prejudice after the manipulations and dependent measures to avoid priming prejudice at the outset. In Study 1, labelling the target as ‘white’ marginally increased prejudice, irrespective of treatment, and in Study 2 neither identity label nor treatment affected prejudice. Thus, we can say that the experience of racial exclusion did not reduce white participants’ levels of prejudice.’	Sample Misinterpretation
Xie *et al.* [50]	‘we found that when people are forming an impression, trait inferences are differently correlated across race and gender in stereotype-consistent ways. Thus, to the extent that physical strength and trustworthiness are negatively associated for Black men but unrelated for White men, sentencing decisions, which are influenced by how trustworthy a target appears, are more likely to be influenced by other attributes (e.g. physical strength) for Black than White male defendants. Given that defendants with faces stereotyped to be crime-congruent are more likely to be found guilty (Macrae & Shepherd, 1989), idiosyncratic stereotypes in impression formation may contribute to systematic discrepancies in conviction rates across groups.’	Population Misinterpretation
Weston *et al.* [51]	‘contrary to our hypotheses, the relationship between work characteristics and sense of purpose was not conditioned by negative and positive forms of work/home spillover. Though further research is needed to examine this lack of moderation, this result suggests the relative stability of the associations between work characteristics and sense of purpose, insofar that their potential benefits do not appear to depend on the employee’s positive or negative perceptions of whether their work and home domains affect one another.’	Population Misinterpretation

#### 4.2.2. Change in prevalence over time

We hypothesized that fewer articles misinterpreted nonsignificance as reflecting no effect in 2015 relative to 2009, and in 2021 relative to 2009 and 2015. Results from multi-level binomial regression analysis suggest that for every 1 article that misinterpreted nonsignificance as reflecting no effect in 2009, 1.20 (95% CI: 0.56–2.58, *p* = 0.642) and 0.78 (95% CI: 0.48–1.28, *p* = 0.329) articles made this interpretation error in 2015 and 2021, respectively. Results also suggest that for every article that misinterpreted nonsignificance as no effect in 2015, 0.65 (95% CI: 0.30–1.38, *p* = 0.260) articles made this interpretation error in 2021. However, all of these effects were statistically nonsignificant.

### 4.3. Exploratory analyses

#### 4.3.1. The use of soft language to infer an effect’s absence

**Figure 2.**
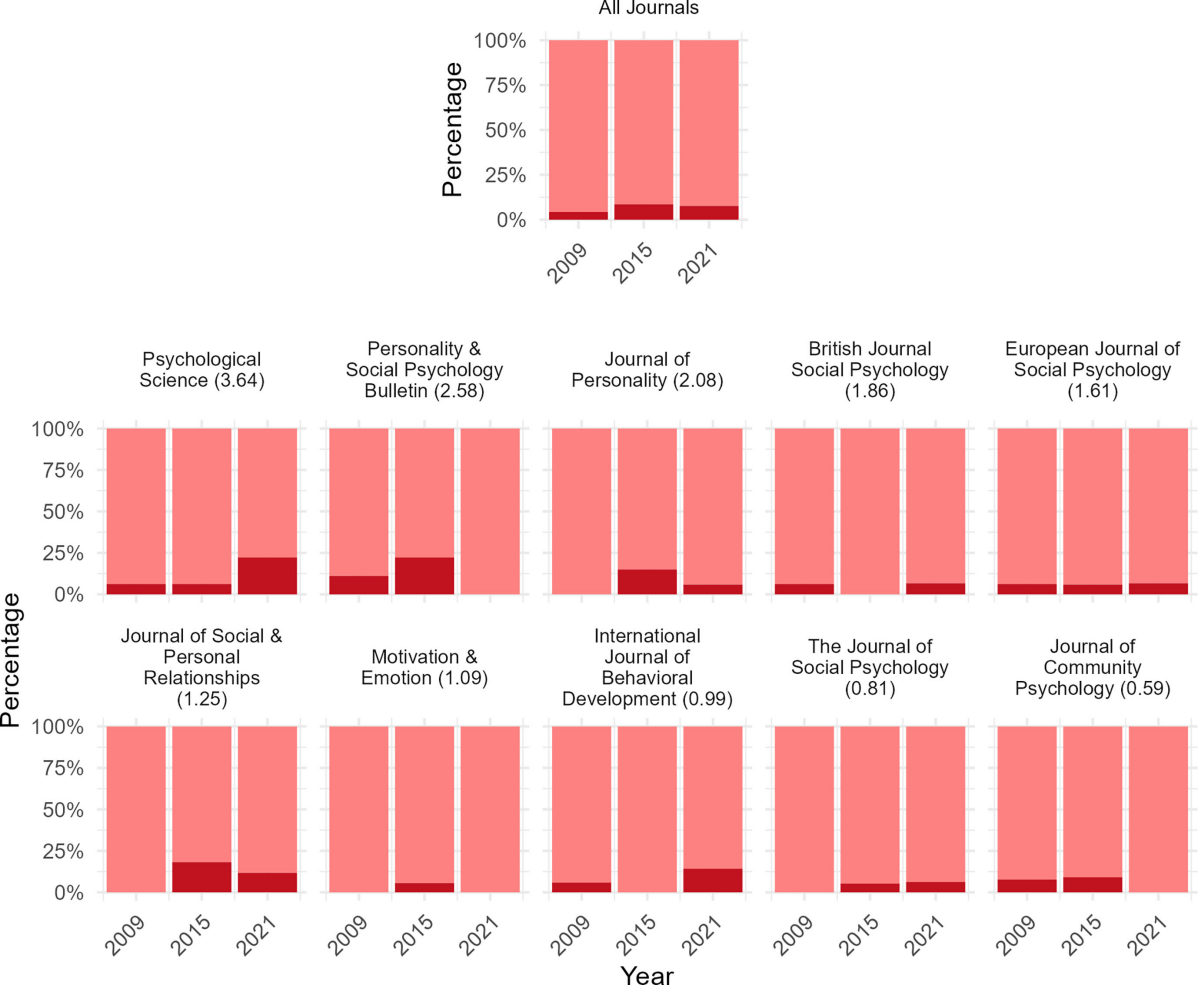
Prevalence of articles that were coded as a misinterpretation from soft, minimal-effect language only, for 10 psychology journals for the years 2009, 2015 and 2021. *Note*. Light bars represent the percentage of articles that misinterpreted nonsignificance that used clear cut ’no effect’ terminology, or a combination of this alongside softer ’minimal effect’ terminology (e.g. ’groups were therefore similar’). Dark bars represent percentage of articles that were coded as a misinterpretation based off softer, minimal effect terminology, only. ‘All Journals’ shows prevalence rates across the 10 journals depicted. The number in brackets after each journal name depicts each journal’s SCImago Journal Rank Indicator. This represents a crude measure of journal quality.

We additionally explored what percentage of psychology articles that misinterpreted nonsignificance as reflecting no effect used soft language to express this (e.g. ‘effects are similar’). See figure 2 for these results across the 10 psychology journals in 2009, 2015 and 2021. Multi-level binomial regression analysis revealed that approximately 6.85% (95% CI: 4.91–9.47%) of all psychology articles that discussed a nonsignificant effect across the examined years may have used soft terminology only. The remainder, used clear-cut ‘no effect’ terminology (e.g. ‘there is no effect’), or a combination of clear-cut and soft terminology. Breaking this down, our models reveal that 4.29% (95% CI: 2.06–8.73%), 8.36% (95% CI: 4.53–14.91%) and 7.36% (95% CI: 3.64–14.29%) of all psychology articles for the year 2009, 2015 and 2021, respectively, may have used soft terminology only.

#### 4.3.2. Nonsignificance explicitly discussed as an effect that could not be found

**Figure 3.**
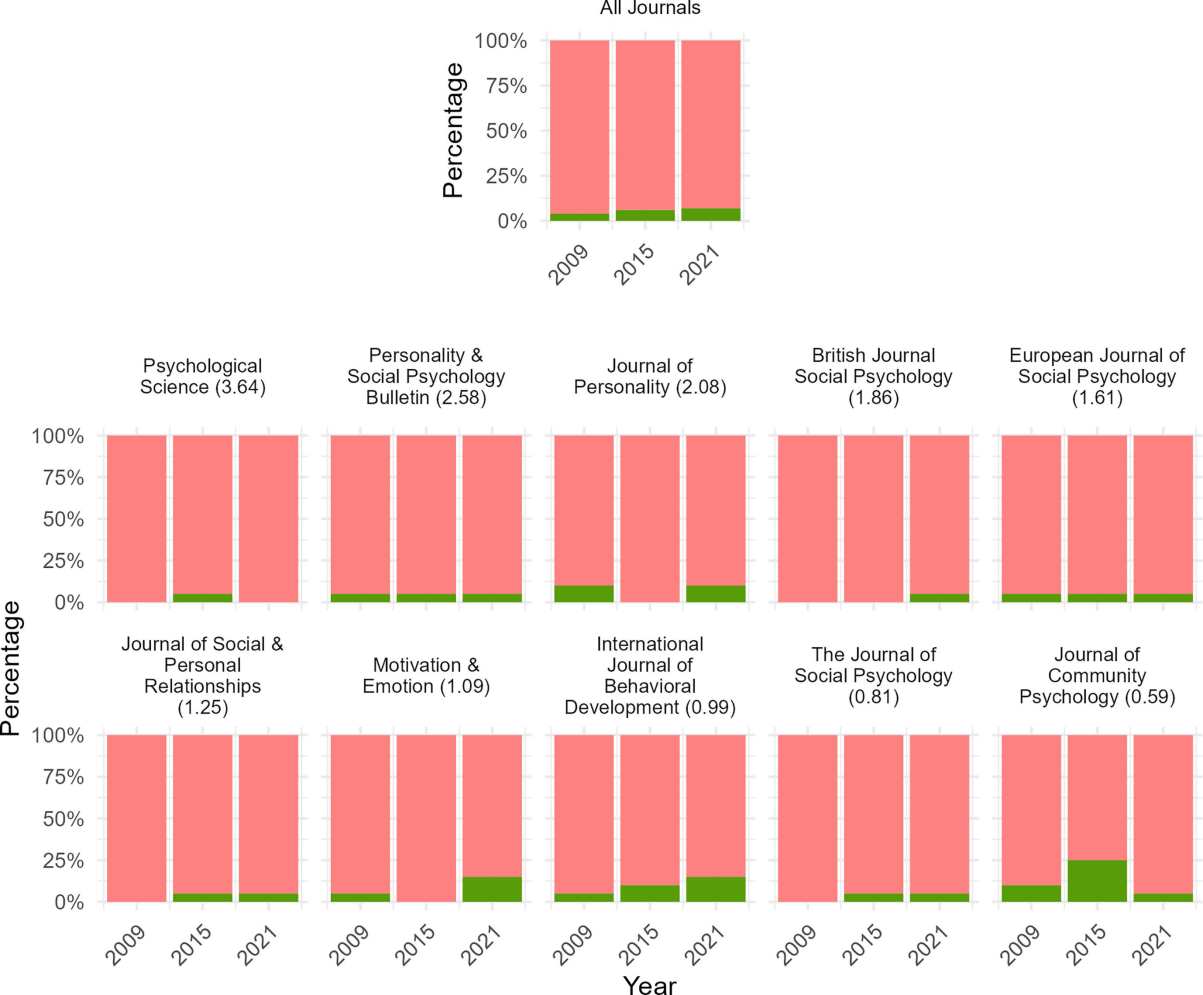
Prevalence of articles that discussed the possibility that nonsignificance could reflect an effect that could not be found, for 10 psychology journals for the years 2009, 2015 and 2021. *Note*. Green and red bars represent the percentage of journals that mentioned a nonsignificant effect and did/did not discuss in detail the possibility that it could be that the effect exists but simply could not be found, respectively. ’All Journals’ shows prevalence rates across the 10 journals depicted. The number in brackets after each journal name depicts each journal’s SCImago Journal Rank Indicator. This represents a crude measure of journal quality.

We further explored how many articles that mentioned a nonsignificant finding discussed in some detail the possibility that it may reflect a true effect that could not be found. See figure 3 for these results across the 10 psychology journals in 2009, 2015 and 2021. Multi-level binomial regression analysis revealed that approximately 5.41% (95% CI: 3.57–8.10%) of all psychology articles over the examined period that mentioned a nonsignificant result explicitly discussed that it may reflect an effect that could not be found. Breaking this down, our models reveal that only 4.02% (95% CI: 2.02–7.83%), 4.76% (95% CI: 2–10.95%) and 7% (95% CI: 4.19–11.47%) of all psychology articles for the year 2009, 2015 and 2021, respectively, may have explicitly discussed one of their nonsignificant findings as though it may be a true effect that could not be found.

#### 4.3.3. Change in prevalence over time

Supplementing our confirmatory examination, we examined whether Population Misinterpretations specifically are becoming less prevalent over time. Multi-level binomial regression analysis suggests that for every one article that made a Population Misinterpretation in 2009, it is likely that 0.77 (95% CI: 0.51–1.16, *p* = 0.207) and 0.75 (95% CI: 0.51–1.13, *p* = 0.167) psychology articles made this interpretation error in 2015 and 2021, respectively. Results also suggest that for every 1 psychology article that made a Population Misinterpretation in 2015, it is likely that 0.65 (95% CI: 0.30–1.38, *p* = 0.260) psychology articles made this interpretation error in 2021. However, all of these effects were statistically nonsignificant.

We also tested our temporal hypotheses concerning the prevalence of nonsignificance misinterpretation, and specifically, Population Misinterpretations, via growth curve analyses, for this approach can grant more statistical power to find temporal effects [52]. Inferential analyses indicated that for every 1 psychology article that misinterprets nonsignificance as reflecting no effect in a particular year, 0.88 (95% CI: 0.69–1.13, *p* = 0.319) psychology articles were predicted to make this misinterpretation error 6 years afterwards. Moreover, for every 1 article that makes a Population Misinterpretation in a particular year, 0.87 (95% CI: 0.71–1.06, *p* = 0.169) psychology articles were predicted to make this misinterpretation error 6 years afterwards. However, all of these effects were statistically nonsignificant.

#### 4.3.4. Are more impactful articles less likely to make this interpretative error?

**Figure 4.**
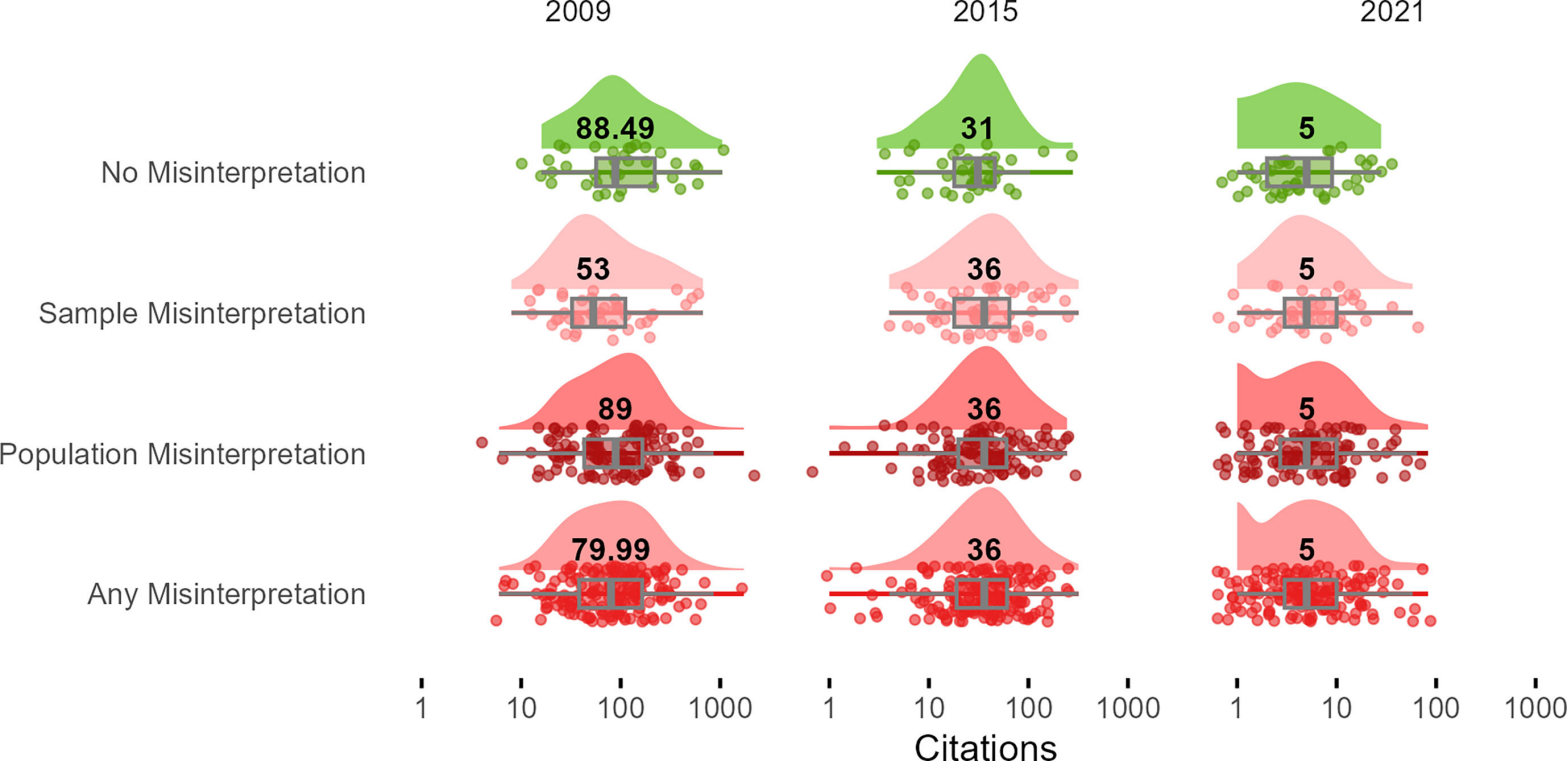
Raincloud plot showing article citation distributions for the year 2009, 2015 and 2021, for articles that did and did not misinterpret nonsignificance as reflecting no effect. *Note*. Density plot, boxplot and datapoints underpinning these plots, depicted. Numbers in density plot refer to median citation number. *X*-axis on logarithmic scale to aid visualization. The term ’Any Misinterpretation’ refers to sample and population misinterpretations combined.

Finally, we explored whether articles that had more citations were less likely to misinterpret an identified nonsignificant result. See figure 4 for a descriptive overview of these results in relation to our sample. Controlling for year of publication, multi-level regression analyses indicate no significant differences in citation number as a result of making or not making the focal interpretation error (b = −17.69, 95% CI: −46.39 to 8.47, *p* = 0.193). Our results indicate similar findings in relation to the prevalence of Population Misinterpretations (b = −0.96, 95% CI: −18.34 to 16.60, *p* = 0.914).

## 5. Discussion

The present study aimed to advance our understanding of how prevalent it is for published psychology articles that employ null hypothesis significance testing to misinterpret a nonsignificant effect as reflecting an effect’s absence, and whether this practice is becoming more or less prevalent over time. This study is important for to efficiently tackle this problem we need a firm understanding of its magnitude, something which we believe is still lacking. Complementing these primary aims, we also examined (i) whether articles that are more impactful (i.e. have more citations) are less likely to make this interpretation error, and (ii) what percentage of articles that mention a nonsignificant finding *discuss* the possibility that it may simply reflect a true effect that could not be found. Overall, amid some nonsignificant results, our findings reveal the scale of this problem is probably *much* worse than previously thought, and that articles very rarely discuss the possibility that nonsignificance may simply reflect an *effect that could not be found* issue.

Specifically, 95% CIs from our analyses indicate that between the years 2009 and 2021 (and arguably in the handful of years before and after, considering the absence of major reform around this period), it is likely that between 76.17% and 84.90% of all psychology articles that mentioned a nonsignificant finding in their discussion section misinterpreted nonsignificance as reflecting the effect’s absence. When considering only Population Misinterpretations (e.g. ‘men and women *do not* differ’), the prevalence rate is probably between 53.93% and 61.82% of all psychology articles that mentioned a nonsignificant finding. If these estimates reflect reality, it would mean that this problem is *much* worse than previously thought. Previous research suggested that between 38% and 72% of articles published during the years examined, and that mentioned a nonsignificant finding, made this error [2,11,17], and that approximately 23% made a Population Misinterpretation [2]. However, because these studies investigated this issue by examining article abstracts only [2], and by coding articles as having made a mistake *only* if very clear-cut terminology was used to indicate this (e.g. ‘There is zero difference between groups’ [2,17]), they very likely underestimated the true magnitude of the issue, the likes of which is arguably best captured (i) by additionally including softer statements that misinterpret nonsignificance, and (ii) by examining an article’s discussion section, because this is where authors are most likely to discuss nonsignificant effects in detail. Because the present study did this, while additionally sampling many more psychology journals (*n* = 10) and articles (*n* = 599) overall, we regard it as more revealing of the issue’s actual prevalence over the period investigated. Another implication that follows if our prevalence estimates are accurate is that published work highlighting this interpretative error (e.g. [21,53]) is not sufficient to tackle this issue. If more is not done, we fear that many readers will continue to take home the problematic message that various effects are completely zero, that many policymakers and funders will continue to draw from a literature characterized by binary ‘effect-no effect’ reasoning, and that many researchers will continue to disregard a huge number of effects that with sufficient statistical power would very likely be found, and would often play an important role in the development of psychological theory.

Some ideas we have for reducing the prevalence of this issue are to better educate researchers on this topic during their formal undergraduate or postgraduate studies. Students have much valuable knowledge to gain over this educational phase to become an effective researcher. Yet, in our minds, it is particularly critical that sufficient time is given to statistical theory and its relation to psychological theory because nearly every article a researcher will read or write from that day forward will demand this foundational knowledge so that findings can be accurately interpreted. The same can not be stated for other important topics covered as part of the formal educational process, thus indicating that we should think seriously about adjusting our teaching and educational priorities. That stated, such educational efforts will only be effective if the educators themselves possess the necessary statistical knowledge. Worryingly, recent research indicates that statistics educators, and the educational resources they, researchers and students use, often perpetuate various misconceptions [54], a problem also identified many decades ago [55].

What also would help reduce the prevalence of this issue is if researchers make it explicit that their nonsignificant findings (i) do not signal that the effect does not exist, and (ii) most likely mean that they just need to get more data to find an effect that may well exist at some magnitude. Unfortunately, the high prevalence rates that our models estimate suggest that researchers should no longer expect that if they state: ‘the effect was nonsignificant’, that readers will interpret this as an effect that could not be *found*. Thus, supplementing nonsignificant findings with a more detailed interpretation will help ensure one’s intended conclusions are correctly conveyed. On this point, our models suggest that only between 3.57% and 8.10% of all published psychology articles that mention nonsignificance in the discussion section explicitly considered the possibility that their nonsignificant finding may reflect a true effect that could not be found. Clearly, there is much room for improvement in this area. Additionally, we call on journals to require authors that have submitted their article to confirm any nonsignificant findings have been explained in such a way that they will not be misinterpreted by readers. Evidence suggesting that research disclosure statements helped increase how many articles specify how their sample size was determined, and why observations were not included in the formal analyses [56], indicates that this approach could also help reduce how often nonsignificance is misinterpreted as an effect’s absence. Alternatively or in addition, handling editors, or editorial staff employed specifically to examine article statistics or rigour (e.g. the Statistics, Transparency, and Rigor Editor at the journal *Psychological Science*), should pay much more attention to how statistics are interpreted, given the high prevalence of mistakes and the considerable impact they can have.

Another solution to this issue could be to better encourage or enable researchers to identify a smallest effect size of interest for their theory. For if researchers leverage a sample that imbues high statistical power to find such an effect magnitude, nonsignificant findings would be more informative than they typically are today (and were over the examined period) where nonsignificance very often results alongside population effect magnitudes that would offer theoretical support [57]. Indeed, researchers could, with a minimum effect size of interest, employ equivalence testing alongside default analyses to test whether the effect is indeed too small to matter [58]. An alternative approach to overcoming this issue could be to encourage researchers to leverage Bayesian analyses. Many researchers have already made the switch from null hypothesis significance testing (NHST), partly because Bayesian analyses enable the modelling of prior information but also because it can provide answers to questions where NHST is unable, like ‘how much more likely is it that the population effect is .10 instead of 0.02, 0.23, or 0.001?’ [59]. Yet, even if equivalence or Bayesian approaches are used, we still advise researchers to not use ‘no effect’ language as a linguistic tool. In short, researchers are simply too susceptible to interpreting ‘no effect’ language as if the effect is indeed completely absent, which is highly unlikely [8].

While our findings also inform to some extent on the percentage of *researchers* in psychology that make this communicative error, and of the percentage of researchers that truly believe nonsignificance reflects an effect’s absence, we caution readers against using the calculated prevalence estimates—which are at the article level—to *accurately* inform on researcher-related questions. It is possible some authors of the examined literature did not know the true meaning of nonsignificance, but that one or more coauthors who led the discussion of the nonsignificant effects *did*, and accordingly communicated the correct conclusion. Vice versa is equally plausible, as is the possibility that researchers know it is inaccurate to leverage ‘no effect’ terminology but do so anyway because it is normal practice within the field. We thus direct readers who would like more exact prevalence estimates in relation to researchers to scenario-based research (e.g. [18]), for it is arguably better placed to provide these insights.

Finally, our study did not find that the prevalence of nonsignificance being interpreted as no effect got better or worse over time. We also did not find that more impactful articles were less likely to make this error. Given this may well reflect a statistical power issue, we welcome efforts that reinvestigate these questions with more data.

### 5.1. Strengths and limitations

Various study strengths have been specified (e.g. number of data points and journals, the inclusion of soft misinterpretations, etc.). However, another is our statement extraction approach, for it ensured author conclusions about a nonsignificant finding were not taken out of context. That is, rather than extracting just a sentence or two for coding, often one or two paragraphs were extracted if the researcher felt like that text connected in some way to the focal nonsignificance discussion (see table 1 for examples). This ensured that the researcher coding the statement had the full context relating to the nonsignificant finding, and thus that the codes applied would match well with any codes applied had the full-text of each article been examined.

Despite this study’s various strengths, it also has some limitations. One we have already touched on is the low inter-rater reliability scores, which culminated because more articles than expected discussed their nonsignificant findings in a vague or convoluted way. We largely addressed this issue by discussing each disagreed-upon statement in detail with a more exacting criteria, prior to applying a final code. Plus, we make all statements and codes available at our OSF page. We thus welcome other research teams—which may have a slightly different view of what constitutes a nonsignificance statement declaring ‘no effect’—to reproduce or replicate our findings. Nevertheless, our study would have been strengthened had we demonstrated greater inter-rater reliability. So future work can achieve this, we recommend that more thorough pilot work is conducted as it will help codes to be applied more objectively to those articles that lay in the grey area around the line of demarcation. Relatedly, we additionally call on researchers to be clearer in their interpretations of their findings. Communicating without care or attention to detail will often engender a variable take-home message in the minds of readers. It can also hinder future research, as demonstrated by Scheel [60], who had to abandon their project due to this very issue.

A second limitation is the relatively low number of articles examined in this study. While this *N* is greater than prior studies that have examined this issue and thus represents a strength, it is also spread across three time points thus increasing the confidence interval width specifying what the likely prevalence rate is for a given year. Also, with a greater sample, we would have been better able to reveal any temporal effects that may exist. We thus recommend that future work investigates these research questions with greater sample sizes. However, given the time and effort that this would entail, we call upon researchers in general to examine whether such tasks can be automated, particularly in light of the considerable utility that large-language models may offer.

## 6. Conclusion

In the present study, our results indicate that between 76.17% and 84.90% of all psychology articles between the years 2009 and 2021 that discussed at least one nonsignificant result may have interpreted nonsignificance as though the effect does not exist. If this estimate reflects reality, it would mean the scale of this problem is much worse than prior research indicated. We thus call on researchers, and stakeholders with an interest in improving psychological science, to take more pronounced steps towards solving this issue.

## Data Availability

All data, R code, and materials are available at our OSF page [61].

## Footnotes

^1^
There were 19 instances where a statement met the criteria in this section, or in the following Population Misinterpretation section, but where the researcher nevertheless felt another code fit better, after considering the broader context provided by the full statement. Thus, two sets of codes were applied to each statement: the first based strictly upon the stated criteria, the second based upon what the researcher felt was most suitable. In this article we only report results from the criteria-based coding. We can confirm that no meaningful differences exist in findings between coding approaches. Nevertheless, we make available results from the alternative coding approach on our OSF page.

^2^
The article-level reliability values are based upon comparing the final allocated codes for the two independently extracted statements.

^3^
Reliability scores in this paragraph are conditional upon whether both researchers agreed that the statement did or did not misinterpret nonsignificance, because researchers only applied codes if such a condition was met. For instance, if one researcher coded a statement as having correctly interpreted significance but the other did not, only the first researcher would code whether the statement contained nonsignificance terminology, etc., and vice versa with the other researcher with respect to soft ‘no effect’ language, etc.

^4^
‘Generally’, because on occasion the broader context provided by the statement informed that an alternative code fit best.
